# Antibiotic Susceptibility Surveillance in the Punjab Province of Pakistan: Findings and Implications

**DOI:** 10.3390/medicina59071215

**Published:** 2023-06-28

**Authors:** Zikria Saleem, Abdul Haseeb, Safa S. Almarzoky Abuhussain, Catrin E. Moore, Sairah Hafeez Kamran, Muhammad Usman Qamar, Aisha Azmat, Giuseppe Pichierri, Fahad Raees, Shahzad Asghar, Amna Saeed, Afreenish Amir, Furqan Khurshid Hashmi, Johanna C. Meyer, Israel Abebrese Sefah, Inaam Ur Rehman, Muhammad Umer Nadeem, Brian Godman

**Affiliations:** 1 Department of Pharmacy Practice, Faculty of Pharmacy, Bahauddin Zakariya University, Multan 60800, Pakistan; 2 Department of Clinical Pharmacy, College of Pharmacy, Umm AL-Qura University, Makkah 21955, Saudi Arabia; 3 Centre for Neonatal and Paediatric Infection, St. George’s University of London, London SW17 0RE, UK; 4 Institute of Pharmacy, Lahore College for Women University, Lahore 54000, Pakistan; 5 Institute of Microbiology, Faculty of Life Sciences, Government College University Faisalabad, Faisalabad 38000, Pakistan; 6 Department of Physiology, College of Medicine, Umm Al-Qura University, Makkah 21955, Saudi Arabia; 7 Microbiology Department, Torbay and South Devon Foundation Trust, Lowes Bridge Torbay Hospital, Torquay TQ2 7AA, UK; 8 Department of Microbiology, Faculty of Medicine, Umm Al-Qura University, Makkah 21955, Saudi Arabia; 9 Department of Pharmacy, University of South Asia, Lahore 54000, Pakistan; 10 Department of Pharmaceutical Sciences, Pak-Austria Fachhochschule, Institute of Applied Sciences and Technology, Haripur 22620, Pakistan; 11 Department of Microbiology, National University of Medical Sciences, Rawalpindi 46000, Pakistan; 12 National Institute of Health, Park Road, Islamabad 45501, Pakistan; 13 Punjab University College of Pharmacy, Faculty of Pharmacy, University of the Punjab, Lahore 54000, Pakistan; 14 Department of Public Health Pharmacy and Management, School of Pharmacy, Sefako Makgatho Health Sciences University, Pretoria 0208, South Africa; 15 South African Vaccination and Immunisation Centre, Sefako Makgatho Health Sciences University, Pretoria 0208, South Africa; 16 Pharmacy Practice Department, School of Pharmacy, University of Health and Allied Sciences, Ho PMB 31, Ghana; 17 Strathclyde Institute of Pharmacy and Biomedical Sciences, Strathclyde University, Glasgow G4 0RE, UK; 18 Centre of Medical and Bio-Allied Health Sciences Research, Ajman University, Ajman 346, United Arab Emirates

**Keywords:** Pakistan, culture and sensitivity, surveillance, national action plans, antimicrobial stewardship programs, audits, AWaRe classification

## Abstract

*Background and Objectives*: The increase in antimicrobial resistance (AMR) across countries has seriously impacted the effective management of infectious diseases, with subsequent impact on morbidity, mortality and costs. This includes Pakistan. Antimicrobial surveillance activities should be mandatory to continually assess the extent of multidrug-resistant bacteria and the implications for future empiric prescribing. The objective of this retrospective observational study was to monitor the susceptibility pattern of microbes in Pakistan. *Materials and Methods*: Clinical samples from seven laboratories in Punjab, Pakistan were collected between January 2018 and April 2019, with Punjab being the most populous province in Pakistan. The isolates were identified and their antimicrobial susceptibility was tested using the Kirby-Bauer disc diffusion assay and micro broth dilution methods. The antibiotics assessed were those typically prescribed in Pakistan. *Results*: In total, 2523 bacterial cultural reports were studied. The most frequently isolated pathogens were *Staphylococcus aureus* (866, 34.3%), followed by *Escherichia coli* (814, 32.2%), Pseudomonas aeruginosa (454, 18.0%) and *Klebsiella pneumoniae* (269, 10.7%). Most pathogens were isolated from pus (1464, 58.0%), followed by urine (718, 28.5%), blood (164, 6.5%) and sputum (81, 3.2%). *Conclusions*: The findings suggest that current antimicrobial options are severally restricted in Pakistan due to the emergence of multidrug-resistant pathogens. This calls for urgent actions including initiating antimicrobial stewardship programs to enhance prudent prescribing of antibiotics. This includes agreeing on appropriate empiric therapy as part of agreed guidelines, in line with the WHO EML and AWaRe book, whilst awaiting culture reports. This is alongside other measures to reduce inappropriate antimicrobial prescribing and reverse the threat of rising AMR.

## 1. Introduction

Antimicrobial resistance (AMR) has become a considerable threat across countries increasing morbidity, mortality and costs, especially among low- and middle-income countries (LMICs) including Pakistan [1,2,3,4,5,6,7,8]. Globally, in 2019, there were 1.27 million deaths directly attributable to AMR, with rates expected to continue rising unless proactively addressed [3]. However, despite affecting all the parameters of healthcare systems, and being increasingly seen as the next pandemic, concerns regarding AMR have remained largely ignored among a number of key stakeholder groups [9,10,11,12,13]. Many drivers contribute to the development of AMR in countries [14,15,16,17]. Key drivers include irrational prescribing and dispensing of antibiotics, including for self-limiting conditions, and, in addition, the absence of fully functioning surveillance systems to monitor the consumption of antibiotics alongside bacterial resistance patterns. In many LMICs, the lack of infection, prevention and control (IPC) groups in hospitals, concerns with sanitation and healthcare infrastructures, lack of knowledge regarding AMR and antibiotics among physicians, as well as their overuse in farming and agriculture, exacerbate AMR [14,15,18,19,20,21,22,23,24,25,26]. Addressing these multiple factors is a challenge to healthcare authorities across different countries, especially among LMICs with more limited resources in terms of available finances, personnel and infrastructure, to undertake routine surveillance of AMR patterns across all sectors of care. One output to coordinate activities within countries has been the development of the Global Action Plan by the World Health Organization (WHO), including the preparedness of countries to tackle AMR [27,28,29]. This has resulted in the development of country-specific National Action Plans (NAPs) including Pakistan [30,31,32,33].

The WHO Global Action Plan (GAP) on AMR declared AMR surveillance as a ‘cornerstone’ to assess the burden of the AMR [34]. The objective of the GAP is to monitor the resistance of specific bacterial pathogens to a number of antibiotics, especially where the irrational use of antimicrobials is appreciably higher compared to other regions [35]. Currently, there are appreciable gaps in knowledge regarding current antimicrobial utilization patterns across sectors as well as resistance rates at a local, regional and national level in a number of countries [32,35]. This is a concern that needs to be addressed, as a lack of knowledge hamper efforts to produce a clear picture of the overall AMR scenario to instigate appropriate actions to adequately address rising concerns with AMR within countries [36]. These concerns are particularly prevalent among LMICs with their resource and personnel issues [32,34,35]. The Global Antimicrobial Resistance Surveillance System (GLASS), developed in response to the WHO Global Action Plan, relies on the NAPs of individual countries [37,38,39]. This surveillance system combines the patient’s epidemiological and laboratory data to understand the extent and impact of AMR on populations, which is increasingly important in Pakistan given rising rates of AMR [40]. However, there are serious concerns with implementing the NAP in Pakistan including resource, knowledge and infrastructure issues [41].

Surveillance to monitor resistance patterns among common human bacterial pathogens is an essential first step to strengthening the AMR evidence base [38]. However, there are concerns with available resources, expertise and infrastructures to fully undertake surveillance in a number of LMICs [11,39,41,42]. This includes Pakistan, where there are appreciable concerns with the current surveillance to fully document AMR patterns across the country, which is currently exacerbated by a lack of requests in hospitals for culture and sensitivity testing; however, progress is being made [6,41,43,44,45]. This is illustrated by very high rates of prescribing of antibiotics in Pakistan among patients admitted to hospitals with COVID-19 during the first five waves at 89.7% of patients, with ‘Watch’ antibiotics being prescribed on 93.4% of occasions adding to AMR [46]. This was despite only a limited number of patients in these tertiary care/teaching hospitals having proven bacterial co-infections (1.14% of admitted patients) or secondary infections (3.14% of patients) [46]. This endorses the need for accurate diagnostic and sensitivity data, even among tertiary hospitals in Pakistan, to improve future antibiotic prescribing and reduce AMR.

One challenge generally among LMICs is the lack of diagnostic facilities and antibiotic sensitivity data, which reduces appropriate prescribing of antibiotics, potentially increasing AMR [47]. This is a concern as the susceptibility patterns of microbes should be increasingly monitored among primary, secondary and tertiary hospitals as well as nationally within LMICs to improve empiric prescribing. This is important given concerns with rising AMR rates in Pakistan, as well as high rates of antimicrobial prescribing among hospitals in Pakistan, often without culture and sensitivity testing [43,44,46,48,49,50], as well as the current absence of national prescribing guidance.

Existing studies performed in Pakistan already show high resistance rates in *Escherichia coli*, *Staphylococcus aureus*, *Acinetobacter baumannii* and *Klebsiella pneumoniae* against cotrimoxazole, third-generation cephalosporins, fluoroquinolones and methicillin [6,44,45]. Consequently, the objective of this study was to build on these findings and describe the susceptibility patterns of bacteria in Pakistan against a range of antibiotics. The findings can subsequently be used to suggest future activities, including introducing additional antimicrobial stewardship programs (ASPs) in hospitals, given increasing concerns about the inappropriate prescribing of antibiotics in hospitals in Pakistan, including high rates of prescribing of ‘Watch’ and ‘Reserve’ antibiotics and their impact on AMR [46,49,50,51,52,53,54,55,56]. This is important since whilst hospitals in Pakistan are currently putting in place the building blocks for ASPs, there are still an appreciable number of activities that are needed before ASPs can be fully implemented in the country [55,57]. The introduction of ASPs in Pakistan, though, is currently hampered by concerns with available resources, as well as the ability of patients to cover the costs of culture and sensitivity testing (CST) themselves, together with treatment costs within public hospitals [50,51,58,59]. The latter must be tackled by the authorities in Pakistan for CST to become routine and for inappropriate prescribing to reduce. Adequate resources must form part of the NAP going forward since it is recognized that ASPs are more difficult to undertake in LMICs due to available personnel and financial issues [41,43,60].

In the first instance, we describe the results of a passive surveillance system from seven public and private sector microbiological laboratories across different cities of Pakistan and the changing antibiotic susceptibility profiles observed across these hospitals. As mentioned, the findings can subsequently be used to guide successful empiric antibiotic treatment in hospitals in Pakistan in the near future as part of planned ASPs to meet the NAP goals. The findings can also be used to push for increased resources among the authorities for increased funding in hospitals to cover the costs of routine CST testing and associated antibiograms.

## 2. Materials and Methods

### 2.1. Study Design and Population

This was a retrospective observational study conducted to observe the patterns of AMR among a range of bacterial isolates. An overview of the methodology is provided in Supplementary Figure S1. The data were collected from both public and private sector microbiological laboratories in different cities across Punjab, Pakistan.

Punjab was chosen for this research project because it is one of the four provinces of Pakistan located in the central-eastern region. It is also the largest province by population, accounting for more than half of the population of Pakistan, as well as the second-largest province of Pakistan by land area [46,49,52,53]. Consequently, if there are considerable issues with AMR in the Punjab province, this will have serious implications for the whole of Pakistan.

A total of eleven pathology and diagnostic laboratories in different areas of Punjab were initially visited. From these, seven laboratories subsequently took part in this study as these laboratories fulfilled the high standards of precision and technique set by the Clinical and Laboratory Standard Institute (CLSI). The consensus-driven medical laboratory standards of CLSI are widely acknowledged as the go-to resources for improving testing quality, safety, and efficiency on an ongoing basis, internationally [61]. The samples were subsequently collected through a non-probability purposive sampling approach in order to select only common pathogens.

All clinical samples arriving at the laboratories between January 2018 and April 2019 were included. These samples included pus (*n* = 1464), urine (*n* = 718), blood (*n* = 164), sputum (*n* = 81), tissue (*n* = 53) and body fluids (*n* = 43), which were collected using aseptic techniques and subsequently transported to the designated microbiology laboratories for analysis.

### 2.2. Inclusion and Exclusion Criteria

The bacterial pathogens were only considered if they were isolated more than 50 times. Patient culture reports with only one particular resistant isolate was included. Patient culture reports with more than one resistant bacterial isolate were excluded from the study because it would interfere with the resistance pattern of each other.

A comprehensive data collection form was developed, keeping in mind the objectives of the study. The following information was included in the data collection form: (a) source of a clinical sample, (b) isolated pathogen and (c) antimicrobial susceptibility pattern.

In the final analysis, four Gram-negative microbes including *Acinetobacter baumannii*, *Escherechia coli*, *Klebsiella pneumoniae*, *Proteus mirabilis* and *Pseudomonas aeruginosa,* and one Gram-positive pathogen, *Staphylococcus aureus*, were included in this study. The data collection form was based on previous studies coupled with the considerable experience of the co-authors, which is similar to other studies conducted by the co-authors [62,63,64,65].

### 2.3. Processing of the Samples and Susceptibility Testing

For blood cultures, 1–3 mL of blood was drawn from children and 5–10 mL of blood was collected from adult patients. The blood culture bottles were placed in an automatic BACTEC 9120 system for up to 5 days to be declared negative. The positive blood samples were subsequently sub-cultured on blood, MacConkey and chocolate agar. The plates were incubated at 35c overnight aerobically.

After overnight incubation, the isolates were preliminary identified based on colonial morphology, culture characteristic, Gram stain and biochemical profile. The Gram-negative isolates were biologically confirmed by API 20 E and 20NE. The Gram-positive bacteria were biochemically confirmed by catalase, coagulase and DNAse test.

The isolates were further analyzed for antimicrobial susceptibility testing by Kirby-Bauer disc diffusion assay as per clinical laboratory standard institute (CLSI) 2020 guidelines. The MRSA was confirmed as per CLSI guidelines. In short, the 0.5McFarland bacterial suspension was swabbed on the Mueller Hinton Agar plate, a cefoxitin disc was placed, and the plate was incubated overnight at 37 °C. The zone of inhibition was measured as described by the CLSI guidelines. MIC test was performed for the Polymyxin B and Colistin by micro broth dilution methods as per CLSI guidelines.

The following antibiotics were assessed and broken down by their ATC classification [66], as they are typically prescribed in Pakistan: doxycycline, tetracycline, minocycline, tigecycline, ampicillin, amoxicillin, penicillin, co-amoxiclav, ampicillin–sulbactam, Piperacillin–tazobactam, oxacillin, piperacillin, cephalexin, cephazolin, cephradine, cefoxitin, cefuroxime, cefaclor, cefotaxime, ceftazidime, ceftriaxone, cefixime, cefoperazone, cefoperazone–sulbactam, cefepime, aztreonam, meropenem, ertapenem, imipenem, erythromycin, clarithromycin, azithromycin, clindamycin, tobramycin, gentamycin, amikacin, ofloxacin, ciprofloxacin, norfloxacin, levofloxacin, moxifloxacin, nalidixic acid, pipemedic acid, vancomycin, teicoplanin, colistin, nitrofurantoin, fosfomycin, linezolid, polymixin-B, streptomycin, rifampin, and co-trimoxazole. The antibiotics included in our study were selected based on the internal policy of the hospitals where the tests were performed. These antibiotics were routinely tested for susceptibility in clinical laboratories as part of their standard practice. Therefore, we included these specific antibiotics in our study to reflect the real-world scenario and provide insights into the susceptibility patterns of the bacteria isolated from the clinical samples. In addition, further analysis using the WHO AWaRe classification [67,68,69,70] and the sample source were conducted. Only the sensitivity of the pathogens against pertinent antibiotics was recorded, with a number of antibiotics inappropriate for the given organism.

### 2.4. Statistical Analysis

SPSS (Statistical Package for the Social Sciences) was used to analyze the data by cross tab which gave selective information about individual susceptibility pattern microbes against various antibiotics.

### 2.5. Ethical Consideration and Patient’s Consent

The study was approved by the Human Ethics Committee of the College of Pharmacy, Bahauddin Zakariya University, Multan, Pakistan (BZU- DEPP-22-830-1005It), with subsequent approval among participating hospitals. The study adheres to the guidelines set forth in the Declaration of Helsinki [71], good clinical practice (GCP) and relevant regulatory requirements.

## 3. Results

### 3.1. Identification of Clinical Pathogens

**Figure 1:**
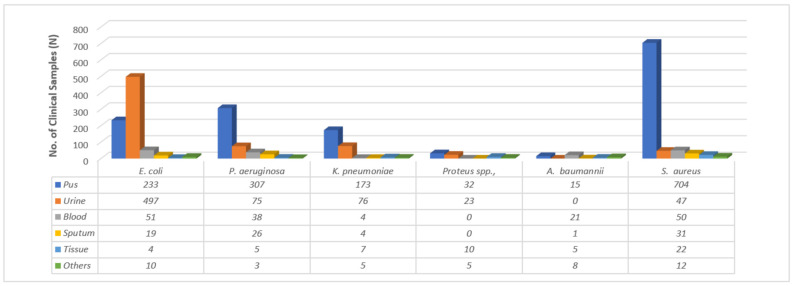
Prevalence of pathogens in different clinical samples.

A total of 2523 cultural reports were studied. Of the 2523 reports, 65.4% were for Gram-negative bacteria and 34.3% were for Gram-positive bacteria. Among Gram-positive bacteria, the most frequently isolated pathogens were *Staphylococcus aureus* (866, 34.3%), while among Gram-negative bacteria, *Escherichia coli* 814, 32.2%), *Pseudomonas aeruginosa* (*454*, 18.0%) and *Klebsiella pneumoniae* (269, 10.7%) were the most common. Most of the pathogens were isolated from pus (1464, 58.0%), followed by urine (718, 28.5%), blood (164, 6.5%) and sputum (81, 3.2%) (Figure 1). Microbes such as *E. coli* (497, 61.10%) were most frequently isolated from urine, whereas *S. aureus* (704, 81.30%) and *P. aeruginosa* (307, 67.60%) were mostly isolated from pus.

### 3.2. Antimicrobial Resistance

Twelve classes of antibiotics were considered for this analysis. These 12 classes included the tetracyclines, penicillins, cephalosporins, carbapenems/monobactams, macrolides, aminoglycosides, fluoroquinolones, glycopeptides, polymixins, nitrofurans, phosphonic antibiotics and oxazolidinones. They were chosen because they constitute the principal antibiotic classes currently being prescribed in hospitals in Pakistan. Out of these 12 classes, if a microbe was resistant to 3 or more classes, it was subsequently considered multidrug-resistant (MDR). If a microbe was resistant to 7 or more classes, it was subsequently considered extensively drug-resistant (XDR).

**Figure 2:**
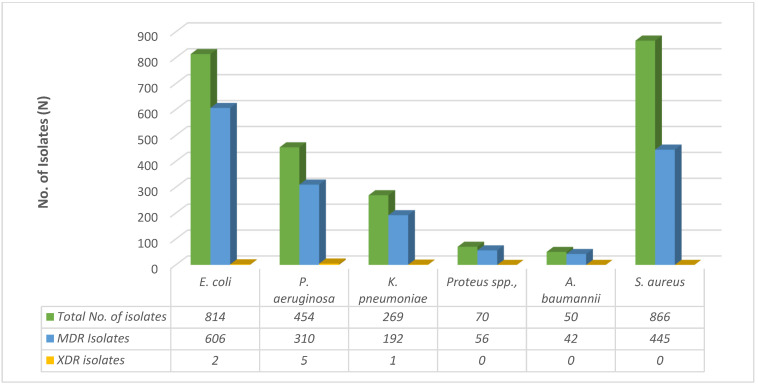
Prevalence of MDR and XDR clinical isolates.

Table 1 and Figure 2 provide the prevalence of MDR and XDR clinical isolates for the various organisms. Out of 814 isolates of *E. coli*, 74.44% (606) were MDR and 0.24% (2) were XDR. Among 454 isolates of *P. aeruginosa*, 68.2% (310) were MDR and 1.6% (5) were XDR. Out of 269 isolates of *K. pneumoniae*, 71.375% (192) were MDR and 0.37% (1) were XDR, and among 866 isolates of *S. aureus*, 51.38% (445) were MDR. In total, there were 2523 isolates analyzed, with 65.43% (1651) identified as MDR and 0.31% (8) identified as XDR. We did not identify any extensively drug-resistant (PDR) bacteria in our study.

### 3.3. Antimicrobial Susceptibility Profile

The five most sensitive antibiotics against *E. coli* were fosfomycin (255/277, 92.1), polymyxin-B (80/87, 92.0%), tigecycline (86/95, 90.5%), imipenem (373/427, 87.4%) and meropenem (542/639, 84.8%). For *Klebsiella pneumoniae*, the five most sensitive antibiotics were polymyxin-B (48/48, 100.0%), tigecycline (16/16, 100.0%), colistin (94/99, 94.9%), imipenem (92/108, 85.2%) and fosfomycin (25/32, 78.1%). For *Pseudomonas aeruginosa*, five highly sensitive antibiotics were polymyxin-B (102/108, 94.4%), colistin (179/209, 85.6%), minocycline (54/70, 77.1%), imipenem (105/149, 70.5%) and tigecycline (18/26, 69.2%). For *Acinetobacter,* the most sensitive antibiotics were polymyxin-B (27/28, 96.4%), colistin (28/35, 80.0%), tigecycline (19/26, 73.1%) and minocycline (21/30, 70.0%). For *S. aureus*, five highly sensitive antibiotics were tigecycline (60/64, 93.8%), minocycline (228/248, 91.9%), linezolid (497/575, 86.4%), fosfomycin (25/29, 86.2%) and amikacin (319/386, 82.6%).

Overall, the most potent antibiotics were polymyxin-B (274/298, 91.9%), tigecycline (208/236, 88.1%), fosfomycin (339/395, 85.8%), minocycline (345/431, 80.0%), imipenem (703/891, 78.9%) and colistin (503/642, 78.3%) (Table 2). Table S1 (Supplementary Materials) contains details on some of the published papers from Pakistan regarding susceptibility profiles of antibiotics to offer further guidance to all key stakeholders in Pakistan to enhance the appropriate prescribing of antibiotics.

## 4. Discussion

We believe this is one of the first studies in Pakistan to comprehensively review antibiotic susceptibility patterns against a range of Gram-positive and Gram-negative pathogens to assist with future antibiotic prescribing. As mentioned, such activities are increasingly important in Pakistan given the current high empiric prescribing across a range of hospitals and ages in Pakistan exacerbated by high patient co-payments including for sensitivity testing [49,50,53,58]. In addition, currently considerable prescribing of ‘Watch’ and ‘Reserve’ antibiotics, reaching 100% of all antibiotics prescribed in some hospitals during the recent pandemic. An appreciable proportion of these will not be appropriate, especially with the WHO target of 60% of prescriptions in hospitals should ideally be ‘Access’ antibiotics [46,49,50,51,52,53,69,72] Alongside this, concerns generally with adherence to recommended guidelines [46,49,50,51,52].

Regular reviews of susceptibility patterns of pathogens are essential to inform empiric antibiotic guidelines at an institutional level and reduce AMR. In the present study, *E. coli* was the most common pathogen isolated, which was resistant to more than one antibiotic, similar to other studies [73,74]. The present data showed that *E. coli* was highly resistant to penicillins, cephalosporins, macrolide and quinolone antibiotics. The resistance of this uropathogenic against quinolone is a concern given the appreciable prescribing or dispensing in Pakistan for this indication [75]. The dispensing of antibiotics without a prescription in most community-acquired infections could be a potential reason for this high resistance, with high rates of dispensing of ‘Watch’ and ‘Reserve’ antibiotics in Pakistan without a prescription [76,77]. Other published studies have also shown increasing resistance against antibiotics with the passage of time [78,79]. In a study involving private hospitals in Lahore, Pakistan, the authors found that out of 93 *Escherichia coli* isolates, 82% were resistant to beta-lactam antibiotics with many resistant to fluoroquinolones and trimethoprim–sulfamethoxazole, which was attributable to antibiotic overuse [80].

Antibiotic sensitivity in commensal *E. coli* has been monitored among healthy Bolivian children. The researchers found that resistance to earlier discovered antibiotics including penicillin and ampicillin was greater than to the relatively newly discovered antibiotics including fluoroquinolones [81]. This has implications for Pakistan.

*Staphylococcus aureus* was the second most common pathogen isolated in our study, highly dominant in pus where they cause skin infections, and is also a major cause of bloodstream infections (BSI) [82,83]. The current data in our study indicated that penicillins, cephalosporins, macrolide and quinolone antibiotics are no longer active against multidrug-resistant pathogens. Even now, these pathogens have developed resistance against linezolid and glycopeptides which also needs addressing going forward [84,85,86].

Similarly, the penicillin class of antibiotics was the first line of defense in the treatment of infections caused by the genus Staphylococcus in Pakistan. However, with the passage of time, increased resistance has been acquired by the microbes resulting in an increased percentage of *methicillin-resistant Staphylococcus aureus* (MRSA) pathogens [87]. Recently acquired resistances of B-lactamases have made treatment difficult for the Gram-negative bacilli and Gram-positive cocci [88]. However, sensitivity patterns are closely related to the person’s age, sex and anti-microbial therapy background. Consequently, physicians should keep these factors in mind when prescribing antibiotics, guided by regularly updated antibiograms in hospitals.

After *E. coli* and *Staphylococcus aureus*, the third most common isolate was *Pseudomonas aeruginosa*, and the resistance pattern of this pathogen was similar. The published data also highlighted the resistance pattern of this microbe against pneumonia [89]. *Klebsiella pneumoniae* is another multidrug-resistant microbe, which is a major cause of community-acquired pneumonia [90,91]. The studies indicated an increase in resistance patterns of this strain, especially against carbapenem, which is another concern going forward [92,93]. However, the present data showed that carbapenems are still active against *Klebsiella pneumoniae*, which is encouraging.

Colistin remains the last resort of antibiotics for life-threatening infections due to carbapenem-resistant pathogens [94] and should be treated as such [68,69]. Similar to other studies, *Enterococci* were also showing resistance to the quinolones [95], whereas penicillins were showing relatively better sensitivities. Moreover, linezolid and glycopeptides were showing full response against *Enterococcus faecalis*. Contrary to other studies, encouragingly no vancomycin-resistant enterococci (VRE) was isolated in this current study [96]. According to a recent study from Pakistan, *E.coli* and *Klebsiella* were the most common pathogens causing urinary tract infections, and aminoglycosides, quinolones and 2^nd^- and 3^rd^-generation cephalosporins are the antibiotics of choice for their treatment [97]. However, AMR was prevalent among *K. pneumoniae,* and *E. coli* carried in the clients attending outpatient clinics in Uganda [98], which is a future concern for Pakistan unless addressed.

AMR has also appreciably impacted the management of healthcare-associated infections (HAIs), which have been the major causes of morbidity and mortality in hospitals across countries [99]. Surveillance of HAIs and AMR are key activities in the management of infection control programs in hospitals globally [100]. Surveillance activities also inform the responsible authorities as well as hospitals to design appropriate protocols for empiric therapy [101]. Consequently, surveillance activities should be regularly performed in hospitals in Pakistan to monitor the extent of multidrug-resistant bacteria in hospitals as well as in the food chain as part of a One Health approach [93,102]. Surveillance activities can be part of future ASPs. However, as mentioned, there have been concerns that ASPs were difficult to implement in LMICs [60]. This is now changing with ASPs being successfully instigated among a range of LMICs [59,103,104,105,106]. We will continue to monitor the situation in Pakistan given the urgency of the situation, especially surrounding the appreciable prescribing of ‘Watch’ antibiotics and their impact on AMR, as well as the ongoing issues with implementing the NAP in the country [41,107].

We are aware of a number of limitations with the study. Firstly, the study design was retrospective and observational, which has inherent limitations, including the inability to establish causality or control for potential confounding variables. This may affect the generalizability and interpretation of the findings. Secondly, this initial study focused on selected cities in the province of Punjab in Pakistan for the reasons stated. Consequently, the findings may not be representative of the entire country. Such studies need to be urgently performed in other provinces of Pakistan to gain a more widespread picture. Thirdly, the study relied on bacterial cultural reports for microbial identification and susceptibility testing. This method may have limitations in terms of sensitivity, specificity and the ability to detect specific resistance mechanisms, particularly in the absence of molecular diagnostic tools. The specific criteria used for selecting the bacterial cultural reports may also introduce potential bias as this method may not capture the full spectrum of microbial isolates and susceptibility patterns in the study population. The absence of molecular diagnostic tools for bacterial detection in the study is also a limitation. These tools can provide more accurate identification and characterization of bacterial strains and resistance genes, which would enhance the understanding of antimicrobial resistance patterns. Despite these limitations, we believe our findings are robust, providing direction to the province to review recent recommendations for the treatment of infectious diseases in the newly launched AWaRe book and adapt accordingly [76].

## 5. Conclusions

In conclusion, based on the findings, the availability of effective antimicrobial treatments is currently appreciably limited in Pakistan due to the rise of multidrug-resistant microorganisms. Consequently, it is increasingly imperative that hospitals in Pakistan implement appropriate ASPs to enhance the appropriate prescribing of antibiotics and reduce AMR. This entails adhering to suitable empiric therapy based on established guidelines aligned with recommendations from the WHO Essential Medicines List (EML) and the AWaRe book, while also including local antibiograms. It is also increasingly essential for clinicians to await culture reports before prescribing antibiotics; however, routine testing requires these costs to be covered by hospitals and not by patients. Alongside this, implementing other measures to enhance appropriate antimicrobial usage to counteract the escalating threat of AMR. We will be following this up in future research projects.

## Figures and Tables

**Table 1:** Resistance of isolates to antibiotic classes.

Isolates	Resistant to 1 Antibiotic Class *n* (%)	Resistant to 2 Antibiotic Classes *n* (%)	Resistant to 3 Antibiotic Classes *n* (%)	Resistant to 4 Antibiotic Classes *n* (%)	Resistant to 5 Antibiotic Classes *n* (%)	Resistant to 6 Antibiotic Classes *n* (%)	Resistant to 7 Antibiotic Classes *n* (%)	Total No. of Isolates
*E. coli*	81 (9.95)	108 (13.26)	221 (27.14)	174 (21.37)	127 (15.60)	61 (7.49)	6 (0.73)	814
*P. aeruginosa*	42 (9.25)	76 (16.74)	122 (26.87)	86 (18.94)	57 (12.55)	23 (5.06)	6 (1.32)	454
*K. pneumoniae*	30 (11.15)	38 (14.12)	72 (26.76)	51 (18.95)	39 (14.49)	23 (8.55)	5 (1.85)	269
*Proteus* spp.	6 (8.57)	5 (7.14)	21 (30)	16 (22.85)	12 (17.14)	6 (8.57)	0 (0)	70
*A. baumannii*	3 (6)	6 (12)	8 (16)	9 (18)	8 (16)	16 (32)	0 (0)	50
*S. aureus*	180 (20.78)	202 (23.32)	190 (21.93)	115 (13.27)	50 (5.77)	40 (4.61)	7 (0.80)	866
Total	342	435	634	451	293	169	24	2523

The principal 7 classes of antibiotics were considered for this analysis. These classes included tetracyclines, penicillins, cephalosporins, carbapenems/monobactams, macrolides, aminoglycosides and fluoroquinolones.

**Table 2:** Frequency (%) of antimicrobial sensitivity of the clinical isolates.

Antibiotics	ATC Classification ^1^	AWaRe Classification ^2^	*A. baumannii*	*E. coli*	*K. pneumoniae*	*Proteus* spp.	*P. aeruginosa*	*S. aureus*	Total
Penicillins									
Penicillin	J01CE01	Access	NA *	NA	NA	NA	NA	153/511 (29.9)	153/579 (26.39%)
Ampicillin	J01CA01	Access	NA	54/543 (9.9%)	NA	5/53 (9.4)	NA	NA	129/1112 (11.61%)
Oxacillin	J01CF04	Access	NA	NA	NA	NA	NA	23/53 (43.4)	23/53 (43.4)
Beta-lactam inhibitors									
Amoxicillin-clavulanate	J01CR02	Access	NA	51/198 (25.8)	23/81 (28.4)	14/44 (31.8)	NA	27/48 (56.3)	115/371 (31.00%)
Ampicillin–Sulbactam	J01CR01	Access	NA	31/93 (33.3)	4/16 (25.0)	0/1 (0)	NA	14/38 (36.8)	49/148 (33.11%)
Piperacillin–Tazobactam	J01CR05	Watch	10/28 (35.7)	415/573 (72.4)	173/231 (74.9)	54/60 (90.0)	241/360 (66.9)	25/47 (53.2)	918/1299 (70.70%)
Cephalosporins									
Cephalexin	J01DB01	Access	NA	7/145 (4.8)	3/37 (8.1)	0/3 (0.0)	NA	60/138 (43.5)	70/323 (21.67%)
Cefazolin	J01DB04	Access	NA	3/44 (6.8)	2/19 (10.5)	1/2 (50.0)	NA	35/67 (52.2)	41/132 (31.06%)
Cephradine	J01DB09	Access	NA	NA	NA	NA	NA	14/42 (33.3)	14/42 (33.3)
Cefoxitin	J01DC01	Watch	NA	NA	NA	NA	NA	243/336 (72.3)	279/400 (69.75%)
Cefuroxime	J01DC02	Watch	NA	74/474 (15.6)	50/201 (24.9)	10/53 (18.9)	NA	69/134 (51.4)	203/862 (23.56%)
Cefaclor	J01DC04	Watch	NA	31/222 (14)	32/91 (35.2)	1/14 (7.1)	NA	35/85 (41.2)	99/412 (24.03%)
Cefotaxime	J01DD01	Watch	0/1 (0.0)	38/171 (22.2)	9/34 (26.5)	0/1 (0.0)	NA	23/72 (31.9)	70/279 (25.09%)
Ceftazidime	J01DD02	Watch	0/22 (0.0)	122/336 (36.3)	67/156 (42.9)	14/25 (56)	122/271 (45.0)	28/86 (32.6)	353/896 (39.42%)
Ceftriaxone	J01DD04	Watch	1/38 (2.6)	95/412 (23.1)	20/86 (23.3)	9/34 (26.5)	NA	111/210 (52.9)	236/780 (30.26%)
Cefixime	J01DD08	Watch	NA	94/437 (21.5)	56/169 (33.1)	11/38 (28.9)	NA	30/86 (34.9)	191/730 (26.16%)
Cefoprazone	J01DD12	Watch	NA	NA	NA	NA	NA	40/64 (62.5)	40/64 (62.5)
Cefoperazone–Sulbactam	J01DD62	Watch	NA	NA	NA	NA	NA	12/29 (41.4)	31/62 (50.0%)
Cefepime	J01DE01	Watch	4/34 (11.8)	56/244 (23.0)	22/56 (39.3)	8/19 (42.1)	48/107 (44.9)	20/69 (29.0)	37/117 (31.7)
Carbapenems									
Imipenem	J01DH51	Watch	10/28 (35.7)	373/427 (87.4)	92/108 (85.2)	25/36 (69.4)	105/149 (70.5)	98/143 (68.5)	703/891 (78.9)
Meropenem	J01DH02	Watch	17/48 (35.4)	542/639 (84.8)	142/203 (70)	48/57 (84.2)	228/346 (65.9)	148/244 (60.7)	1125/1537 (73.12%)
Etrapenem	J01DH03	Watch	NA	96/123 (78.0)	27/42 (64.3)	14/19 (73.7)	NA	2/4 (50.0)	156/225 (69.3)
Macrolides									
Erythromycin	J01FA01	Watch	NA	NA	NA	NA	NA	296/559 (53)	296/559 (53)
Clarithromycin	J01FA09	Watch	NA	NA	NA	NA	NA	122/255 (47.8)	122/255 (47.8)
Azithromycin	J01FA10	Watch	NA	NA	NA	NA	NA	36/128 (28.1)	36/128 (28.1)
Clindamycin	J01FF01	Access	NA	NA	NA	NA	NA	412/665 (62.0)	412/665 (62.0)
Aminoglycosides									
Gentamicin	J01GB03	Access	7/38 (18.4)	291/518 (56.2)	75/149 (50.3)	22/48 (45.8)	120/225 (53.3)	331/495 (66.9)	846/1473 (57.4)
Amikacin	J01GB06	Access	16/41 (39)	498/631(78.9)	141/211 (66.8)	40/58 (69)	247/356 (69.4)	NA	942/1297 (72.63%)
Tobramycin	J01GB01	Watch	8/23 (34.8)	107/233 (45.9)	61/111 (55)	14/23 (60)	137/227 (60)	NA	327/617 (52.97%)
Fluoroquinolones									
Ciprofloxacin	J01MA02	Watch	7/32 (21.9)	164/555 (29.5)	81/164 (49.4)	27/47 (57.4)	150/288 (52.1)	250/580 (43.1)	679/1666 (40.8)
Levofloxacin	J01MA12	Watch	11/23 (47.8)	143/374 (38.2)	59/152 (38.8)	20/39 (51.3)	117/215 (54.4)	134/245 (54.7)	484/1048 (46.2)
Norfloxacin	J01MA06	Watch	NA	90/344 (26.2)	46/118 (39.0)	11/23 (47.8)	73/182 (40.1)	33/116 (28.4)	253/783 (32.36%)
Ofloxacin	J01MA01	Watch	NA	62/256 (24.2)	50/83 (60.2)	1/9 (11.1)	56/133 (42.1)	25/68 (36.8)	194/549 (35.34%)
Moxifloxacin	J01MA14	Watch	NA	NA	6/34 (17.6)	NA	NA	56/107 (52.3)	62/141 (44.0%)
Nalidixic acid	J01MB02	NA	NA	48/294 (16.3)	24/104 (23.1)	2/20 (10.0)	NA	NA	74/418 (17.70%)
Tetracyclines									
Doxycycline	J01AA02	Access	1/11 (9.1)	82/191 (42.9)	9/20 (45.0)	3/5 (60.0)	NA	69/126 (54.8)	164/353 (46.45)
Tetracycline	J01AA07	Access	2/16 (12.5)	77/258 (29.8)	57/120 (47.5)	NA	NA	194/399 (48.6)	339/825 (36.63)
Minocycline	J01AA08	Watch	21/30 (70.0)	25/52 (48.1)	12/19 (63.2)	5/12 (41.7)	NA	228/248 (91.9)	291/361 (80.61%)
Tigecycline	J01AA12	Reserve	NA	86/95 (90.5)	NA	NA	NA	60/64 (93.8)	146/159 (91.82%)
Carb–Monobactams									
Aztreonam	J01DF01	Reserve	NA	35/100 (35)	1/3 (33.3)	NA	14/42 (33.3)	0/6 (0.0)	50/151 (33.1)
Glycopeptide									
Vancomycin	A07AA09	Watch	NA	NA	NA	NA	NA	513/737 (69.6)	513/737 (69.6)
Tecoplanin	J01XA02	Watch	NA	NA	NA	NA	NA	73/125 (58.4)	73/125 (58.4)
Polymixin									
Colistin	A07AA10	Reserve	28/35 (80.0)	177/231 (76.6)	94/99 (94.9)	NA	179/209 (85.6)		489/601 (81.36%)
Polymixin-B	A07AA05	Reserve	27/28 (96.4)	80/87 (92.0)	48/48 (100.0)	NA	102/108 (94.4)	NA	265/288 (92.01%)
Phosphonic									
Fosfomycin	J01XX01	Watch	NA	255/277 (92.1)	25/32 (78.1)	NA	NA	NA	280/309 (90.61%)
Nitrofuran									
Nitrofurantoin	J01XE01	Access	3/5 (60.0)	171/205 (83.4)	14/18 (77.8)	NA		25/34 (73.5)	222/283 (78.4)
Oxazolidinones									
Linezolid	J01XX08	Reserve	NA	NA	NA	NA	NA	497/575 (86.4)	497/575 (86.4)
Sulphonamide									
Trimethoprim–sulfamethoxazole	J01EE01	Access	1/32 (3.1)	68/359 (18.9)	28/92 (30.4)	4/25 (16.0)	NA	78/327 (23.8)	179/835 (21.44%)

NA*: not applicable, ATC classification ^1^: Anatomical Therapeutic Chemical (ATC) classification: AWaRe classification ^2^: ‘A’ = access antibiotics, ‘W’ = watch antibiotics and ‘R’ = reserve antibiotics [66,67,68].

## Data Availability

Additional data is available upon reasonable request from the corresponding authors.

## Supplementary Materials

The following supporting information can be downloaded at: https://www.mdpi.com/article/10.3390/medicina59071215/s1, Supplementary Table S1: Published paper from Pakistan on susceptibility profiles of antibiotics. This contains additional references [97,108,109,110,111,112].
