# Supplementary material for: Antibiotic Susceptibility Surveillance in the Punjab Province of Pakistan: Findings and Implications
Source: Medicina (Kaunas). 2023 Jun 28;59(7):1215. doi: 10.3390/medicina59071215 (PMC10383515; doi:10.3390/medicina59071215)
Supplement: Supplementary file 1 [file medicina-59-01215-s001.zip › medicina-2444495-supplementary.pdf]

Reference	Author & year	Location	Healthcare settings	Sample	Microbes	Most resistant antibiotics	Most sensitive antibiotics	Inference
[108]	Latif, 2009	Lahore	Microbiology Section, Department of Pathology, Services Institute of Medical Sciences (SIMS)	Blood	Staph. Aureus, Klebsiella Spp. and E. coli, Pseudomonas Spp. and Acinetobacter Spp	Oxacillin, 3rd generation Cephalosporins, Carbapenems	-	Multidrug resistant pathogens cause longer hospital stays, expensive drugs, higher mortality, requiring an action plan.
[109]	Samad, 2017	Peshawar	Northwest General Hospital and Research Centre	Sputum	Pseudomonas aeruginosa	Cefoperazone, sulbactam (16.9%)	Amikacin (92.86%), Meropenem (91.55%),	P. aeruginosa, commonly isolated, is increasingly resistant to commonly used antimicrobials.
[110]	Mansoor, 2009	Karachi	Department of ENT, Karachi Medical and Dental College & Abbasi Shaheed Hospital	Pus from discharging ears	S. aureus, Pseudomonas aeruginosa	Ceftriaxone	Amikacin, Ceftazidime, Ciprofloxacin	The resistance to ceftriaxone is very high.
[90]	Zafar, 2016	Islamabad	Pathology Department, Al Nafees Medical College	Urine	E. coli, K. pneumoniae, P. aeruginosa	-	Quinolones, aminoglycosides, cephalosporins	Quinolones, aminoglycosides, and cephalosporins are the preferred drugs for treating urinary tract infections.
[111]	MS Khan, 2009	Pakistan	public sector intensive care unit in Pakistan	Blood	Acinetobacter spp., P. aeruginosa, K. pneumoniae	Tetracycline, Tazobactam/Piperacillin, Meropenem, Polymixin, Ofloxacin	Ceftriaxone, Gentamicin, Ceftazidime	Supplement infection control education with a hospital system that facilitates surveillance programs and practices for effective infection control.
[112]	Masood, 2022	Pakistan	National Institute of Health	Sputum	K. pneumoniae, Acinetobacter, Pseudomonas spp	Imipenem, Meropenem, Gentamicin	Ceftazidime, Ciprofloxacin, Tetramycin	Klebsiella pneumoniae and Acinetobacter sp. frequently appear in sputum cultures and show resistance to certain antibiotics.
